# Supplementary material for: Genetic diversity of CHC22 clathrin impacts its function in glucose metabolism
Source: eLife. 2019 Jun 4;8:e41517. doi: 10.7554/eLife.41517 (PMC6548504; doi:10.7554/eLife.41517)
Supplement: Transparent reporting form [file elife-41517-transrepform.docx]

***eLife’s* transparent reporting form**

We encourage authors to provide detailed information *within their submission* to facilitate the interpretation and replication of experiments. Authors can upload supporting documentation to indicate the use of appropriate reporting guidelines for health-related research (see [EQUATOR Network](http://www.equator-network.org/%20)), life science research (see the [BioSharing Information Resource](https://biosharing.org/)), or the [ARRIVE guidelines](http://www.plosbiology.org/article/info:doi/10.1371/journal.pbio.1000412) for reporting work involving animal research. Where applicable, authors should refer to any relevant reporting standards documents in this form.

If you have any questions, please consult our Journal Policies and/or contact us: [editorial@elifesciences.org](mailto:editorial@elifesciences.org).

**Sample-size estimation**

- You should state whether an appropriate sample size was computed when the study was being designed
- You should state the statistical method of sample size computation and any required assumptions
- If no explicit power analysis was used, you should describe how you decided what sample (replicate) size (number) to use

Please outline where this information can be found within the submission (e.g., sections or figure legends), or explain why this information doesn’t apply to your submission:

No power analysis was performed to determine sample size in experiments 7 and 8 because experimental variability couldn’t be estimated due to the novelty of these experiments. For population genetics and phylogenetics analyses, we used all available (and relevant) samples, and therefore the limit is given by the currently publicly available data sets.

**Replicates**

- You should report how often each experiment was performed
- You should include a definition of biological versus technical replication
- The data obtained should be provided and sufficient information should be provided to indicate the number of independent biological and/or technical replicates
- If you encountered any outliers, you should describe how these were handled
- Criteria for exclusion/inclusion of data should be clearly stated
- High-throughput sequence data should be uploaded before submission, with a private link for reviewers provided (these are available from both GEO and ArrayExpress)

Please outline where this information can be found within the submission (e.g., sections or figure legends), or explain why this information doesn’t apply to your submission:

Figure 7 C, D & E: the number of cells analyzed ranges from 20 (CHC17) to 47 (CHC22M), split into 5 independent biological replicates.

Figure 8 A & B: 10,000 cells analyzed per FACS run. The number of FACS runs performed per experimental conditions: 4 to 8, split into 4 independent biological replicates. Population genetics and phylogenetics analyses did not require any replicate, although control genes were used when appropriate.

**Statistical reporting**

- Statistical analysis methods should be described and justified
- Raw data should be presented in figures whenever informative to do so (typically when N per group is less than 10)
- For each experiment, you should identify the statistical tests used, exact values of N, definitions of center, methods of multiple test correction, and dispersion and precision measures (e.g., mean, median, SD, SEM, confidence intervals; and, for the major substantive results, a measure of effect size (e.g., Pearson's r, Cohen's d)
- Report exact p-values wherever possible alongside the summary statistics and 95% confidence intervals. These should be reported for all key questions and not only when the p-value is less than 0.05.

Please outline where this information can be found within the submission (e.g., sections or figure legends), or explain why this information doesn’t apply to your submission:

In the figure legends for Figures 7 and 8, we specify the type of results plotted with assessment of dispersion as well as the statistical test used and the p value below which differences are considered significant.

More specifically:

In Figures 7C, results plot normalized fluorescence intensities values (+/- SEM) across time. Fluorescence intensities of the CHC17-GFP group were normalized so that the fluorescence intensity values for the longest times of recording would reach 100%. Then the fluorescence intensities of the other experimental groups (CHC22M-GFP and CHC22V-GFP) were adjusted relative to the CHC17 group for comparison.

In Figure 7D, area under the curve values are plotted for each experimental group, as well as the averaged value +/- SEM.

In Figure 7E, the mobile fraction (M_f_) was calculated for each FRAP measurement following the formula M_f_= (F_∞_-F_o_)/(F_i_-F_o_), where F_∞_ is the fluorescence intensity in the bleached region after recovery, F_i_ is the fluorescence intensity before bleaching and F_o_ is the fluorescence intensity immediately recorded after the bleach. Then the average values were determined and plotted +/-SEM.

In Figure 8A, averaged values of fluorescence intensity ratios of surface (HA-tag) GLUT4 over total (mCherry-tag) GLUT4 were plotted +/- SEM for each experimental group.

In Figure 8B, the averaged values of total GLUT4 (mCherry) fluorescence intensities for each experimental group.

In these Figures, three experimental groups were compared in Figures 7 and 8. We performed analyzes of variance (ANOVA) followed with Tukey’s multiple comparison test. For Figure 8A, Student’s t-tests were performed to determine the effect of insulin stimulation on GLUT4 translocation to the surface in cells (no insulin vs. insulin). Statistical significance was reached when p≤0.05.

For phylogenetics analysis, the difference between evolutionary rates was tested using a Kolmogorov-Smirnov test. For population genetics analyses, statistical significance was assessed using a control distribution and by calculating empirical percentile ranks. Difference in frequency between hunter-gatherers and farmers was assessed by calculating the 95% highest posterior density credible interval with a noninformative Beta prior distribution.

(For large datasets, or papers with a very large number of statistical tests, you may upload a single table file with tests, Ns, etc., with reference to sections in the manuscript.)

**Group allocation**

- Indicate how samples were allocated into experimental groups (in the case of clinical studies, please specify allocation to treatment method); if randomization was used, please also state if restricted randomization was applied
- Indicate if masking was used during group allocation, data collection and/or data analysis

Please outline where this information can be found within the submission (e.g., sections or figure legends), or explain why this information doesn’t apply to your submission:

For results shown in Figures 7C-E, groups were established based on treatments (transfection with cDNA coding for CHC17-GFP, CHC22M-GFP or CHC22V-GFP)

For Figure 8, experimental groups were established based on siRNA and cDNA transfections, insulin stimulation (A) and fluorescence intensities (B)). Human populations from the 1000 Genomes Project were allocated to meta-populations (when relevant) using the classification provided by the 1000 Genomes Project Consortium.

**Additional data files (“source data”)**

- We encourage you to upload relevant additional data files, such as numerical data that are represented as a graph in a figure, or as a summary table
- Where provided, these should be in the most useful format, and they can be uploaded as “Source data” files linked to a main figure or table
- Include model definition files including the full list of parameters used
- Include code used for data analysis (e.g., R, MatLab)
- Avoid stating that data files are “available upon request”

Please indicate the figures or tables for which source data files have been provided:

We provide source data for Figure 7C-E and Figures 8A-B. Scripts used are available at https://github.com/mfumagalli/Clathrins_evolution.
